# Antibacterial and Antioxidant Potential of Silver Nanoparticles Biosynthesized Using the Spruce Bark Extract

**DOI:** 10.3390/nano9111541

**Published:** 2019-10-30

**Authors:** Corneliu Tanase, Lavinia Berta, Năstaca Alina Coman, Ioana Roșca, Adrian Man, Felicia Toma, Andrei Mocan, Alexandru Nicolescu, László Jakab-Farkas, Domokos Biró, Anca Mare

**Affiliations:** 1 Department of Pharmaceutical Botany, “George Emil Palade” University of Medicine, Pharmacy, Sciences and Technology of Târgu Mureș, 38 Gheorghe Marinescu Street, 540139 Târgu Mureș, Romania; rosca_ioana01@yahoo.com; 2 Department of General and Inorganic Chemistry, “George Emil Palade” University of Medicine, Pharmacy, Sciences and Technology of Târgu Mureș, 38 Gheorghe Marinescu Street, 540139 Târgu Mureș, Romania; lavinia.berta@umfst.ro; 3 Department of Microbiology, “George Emil Palade” University of Medicine, Pharmacy, Sciences and Technology of Târgu Mureș, 38 Gheorghe Marinescu Street, 540139 Târgu Mureș, Romania; adrian.man@umfst.ro (A.M.); felicia.toma@umfst.ro (F.T.); anca.mare@umfst.ro (A.M.); 4 Department of Pharmaceutical Botany, ”Iuliu Hațieganu” University of Medicine and Pharmacy, 23 Gheorghe Marinescu Street, 400337 Cluj-Napoca, Romania; Mocan.Andrei@umfcluj.ro (A.M.); alexandru_s_nicolescu@yahoo.com (A.N.); 5 Laboratory of Chromatography, ICHAT, University of Agricultural Sciences and Veterinary Medicine, 400372 Cluj-Napoca, Romania; 6 Faculty of Engineering, Sapientia University, 540485 Târgu Mureș, Romania; jflaci@ms.sapientia.ro (L.J.-F.); dbiro@ms.sapientia.ro (D.B.)

**Keywords:** antioxidant, antibacterial, spruce bark, silver nanoparticles

## Abstract

Biosynthesized silver nanoparticles (AgNPs) are widely used in Pharmacy and Medicine. In particular, AgNPs synthesized and mediated by plant extracts have shown topossess several biological activities. In the present study, AgNPs were synthesized using *Picea abies* L. stem bark extract as reducing agent. Factors, such as metal ion solution, pH, and time, which play a role in the AgNPs synthesis, were assessed. The synthesized AgNPs were characterized by Ultraviolet-Visible Spectrometry, Fourier transform infrared spectroscopy, and Transmission Electron Microscopy (TEM). Further, the study has been extended to evaluate the antimicrobial and antioxidant activity of AgNPs. The broad peak obtained at 411–475 nm (UV-Vis spectroscopy), and the color change pattern, confirmed the synthesis of AgNPs. TEM results showed spherical or rarely polygonal AgNPs with an average size of 44 nm at pH = 9. The AgNPs showed antioxidant activity and antibacterial effect against human pathogenic Gram-positive and Gram-negative bacteria. The results show that spruce bark extract is suitable for obtaining AgNPs, with antibacterial and antioxidant activity.

## 1. Introduction

The bark of woody vascular plants has an essential role in plant protection [1,2]. The bark is considered to be a forest waste. After wood processing, a significant amount of waste bark is obtained. Thus, the waste bark can be a cheap source of bio-compounds with high recovery and functionalization potential [2,3]. The bark extracts can have an important biological activity (antioxidant, antibacterial, antitumoral, etc.) [1,4]. Our previous work demonstrated the antibacterial and antitumoral activity of waste bark extracts [5,6,7].

The main biomolecules identified in spruce bark extract are catechin, vanillic acid, gallic acid isorhapontin, piceid, and astringin [4,8]. These compounds are valuable for their antioxidant and antibacterial activity [5,8]. Biomolecules, such as proteins, glycoproteins, lipids, fatty acids, phenols, flavonoids, and sugars, have substantial control of free radical formation [9]. For example, flavonoids can chelate and actively reduce metal ions in nanoparticles, due to numerous hydroxyl and carbon groups [10]. In an experimental study, it was observed that AgNPs synthesized using water extract of garlic, ginger, and cayenne pepper, showed a reduction activity of 2,2-diphenyl-1-picrylhydrazyl and 2,2’-azino-bis (3-ethylbenzothiazoline-6-sulphonic acid) radicals [11].

A group of researchers [12] synthesized AgNPs by reducing Ag^+^ ions with onion extract (*Allium cepa*). *Klebsiella pneumoniae, Proteus vulgaris,* and *Serratia marcescens* were resistant to the onion extract. However, their growth was inhibited by AgNPs synthesized with the same extracts. In the presence of AgNPs, *Pseudomonas aeruginosa* ATCC 10145 and *Bacillus subtilis* NCTC 10400 presented the largest inhibition diameter [12]. In another study coordinated by Patil et al. [13], AgNPs were synthesized from *Lantana camara* leaves. They showed significant antimicrobial activity against *Staphylococcus aureus, Pseudomonas aeruginosa,* and *Escherichia coli*, compared to the standard Ciprofloxacin. The results were compared with the antibacterial activity of the petroleum ether extract and the essential oil isolated from the *Lantana camara* L. leaves. The antibacterial activity of AgNPs was higher compared to the petroleum ether extract and the essential oil of the leaves of *L. camara* L., showing that AgNPs have dose-dependent membrane permeability. Furthermore, the comparison study reveals that AgNPs size and morphology affects the antibacterial activity [13].

Based on the literature data, the main objectives of the current study are: Biosynthesis of AgNPs using polyphenolic spruce bark extract (SBE), with a previously developed technique [14,15]; the determination of the influence of different factors on the biosynthesis processes (metallic salt, time and pH); the characterization of biosynthesized AgNPs by specific assays: Ultraviolet-Visible Spectrometry (UV-Vis), Fourier transforms infrared spectroscopy (FTIR), and Transmission Electron Microscopy (TEM); and the evaluation of antioxidant and antibacterial activity for biosynthesized AgNPs.

## 2. Materials and Methods

### 2.1. Reagent and Materials

The spruce bark waste comes from a wood processing company from Vatra Dornei, Romania. The waste was dried under room temperature, aeration conditions, and ground using a grinding mill type Veb Nossener Maschinenbau 1980 (Leipzig, Germany). Chemical reagents were acquired from Sigma-Aldrich (Steinheim, Germany) as silver nitrate (AgNO_3_), silver acetate (AgC_2_H_3_O_2_), Folin–Ciocâlteu reagent and potassium bromide (KBr). ABTS was acquired from Merck Company (Darmstadt, Germany), DPPH and 6-hydroxy-2,5,7,8-tetramethylchroman-2-carboxylic acid (Trolox) were acquired from Alfa-Aesar (Karlsruhe, Germany).

The following bacterial strains from the Microbiology Laboratory of UMPhST Târgu Mureș were used: *Staphylococcus aureus* ATCC 25923, methicillin-resistant *S. aureus* (MRSA) ATCC 43300, *E. coli* ATCC 25922, *Klebsiella pneumoniae* ATCC 700603, *P. aeruginosa* ATCC 27853.

### 2.2. Preparation and Characterization of Extract

The spruce bark extract was obtained by placing 10 g of spruce bark in an Erlenmeyer with 100 mL of distilled water. The Erlenmeyer was then inserted into the ultrasonic water bath (Professional Ultrasonic Cleaner MRC (Beijing, China): AC 150 H, 150 W, 40 kHz, heating power 300 W) at 70 °C, for 30 min. The obtained solution was filtered, centrifuged, and stored at 4 °C until further use.

The determination of the total polyphenol content (TPC) was performed spectrophotometrically, using the Folin-Ciocâlteu method [16]. The TPC of the filtered extract was assessed based on the gallic acid calibration curve, the final results being expressed in mg GAE/g biomass.

### 2.3. Synthesis of Silver Nanoparticles

In a previous work (yet unpublished data), the biosynthesis method was optimized by evaluating the influence of the concentration of silver nitrate or the ratio between the extract and the saline solution [14]. The silver solutions were prepared from silver nitrate (AgNO_3_) and silver acetate (AgC_2_H_3_O_2_). Briefly, 10 mL of spruce bark extract was mixed with 90 mL of silver nitrate/silver acetate (1 mM). The solutions were brought to pH = 4 using HNO_3_ and to pH = 9 using NaOH. The biosynthesis took place on an ultrasonic bath (60 °C, 3 h) until the color transformation indicated that the AgNPs synthesis is complete. Finally, four test solutions were obtained: AgNIT4—spruce bark extract, pH = 4, AgNO_3_; AgNIT9—spruce bark extract, pH = 9, AgNO_3_; AgACE4—spruce bark extract, pH = 4, AgC_2_H_3_O_2_; AgACE9—spruce bark extract, pH = 9, AgC_2_H_3_O_2_.

### 2.4. Characterization of Silver Nanoparticles Using Spruce Bark as Reducing Agent

For UV-Vis analysis, the measurements were performed at 1 nm resolution, and the wavelength range between 350 and 700 nm (Analytik Jena Specord 210 Plus190—UV-Vis-1100 nm, Germany). The samples were measured at the initial moment and after 1, 2, and 3 hours.

FTIR analysis involves mixing the AgNPs powder with KBr in agate mortar (1:100). The spectra for spruce bark extract and tested solutions were observed with FTIR Thermo Nicolet 380 Spectrophotometer (Thermo Scientific, Waltham, USA, range 4000–400 cm^−1^).

The morphology and size of the synthesized AgNPs were characterized by transmission electron microscopy using a JEOL 100 U TEM microscope (Japan Electron Optics Laboratory, Tokyo, Japan) at 100 kV on nitrocellulose substrates. The surface-enhanced Raman spectroscopy was recorded with Raman Systems R3000 CN, equipped with a 785 nm diode. The laser power was 200 mV, and the integration time was 30 s. The images were processed with ImageJ program (Wayne Rasband, MD, USA).

### 2.5. In Vitro Antioxidant Activity

The capacity to scavenge the free radical 2,2-diphenyl-1-picrylhydrazyl (DPPH) was performed by using a SPECTRO star Nano microplate reader (BMG Labtech, Offenburg, Germany) and monitored according to the method described by Martins et al. (2015) [17] with some modifications. The results were expressed as Trolox equivalents per gram of dry weight (mgTE/g dw). The radical scavenging activity of the AgNPs against the stable synthetic 2,2′-azino-bis (3-ethylbenzothiazoline-6-sulphonic acid (ABTS) radical cation was measured using a method previously described [18]. The final results were expressed as mg TE/g dw.

### 2.6. Antibacterial Activity

In order to determine the minimal inhibitory concentration (MIC) of the obtained AgNPs against tested bacterial strains, we used the microdilution method, as previously described [6]. For the solutions with a high degree of turbidity, resazurin was used as an indicator of bacterial growth. A color change of resazurin from purple to pink, indicated bacterial growth. MIC was considered in the last well by which the resazurin color did not change.

From each well where no bacterial growth was observed at the MIC method, 1 µl of suspension was spot-inoculated on blood agar, with a calibrated bacteriological inoculation loop. Minimal bactericidal concentration (MBC) was considered the first concentration where no bacterial growth was observed on blood agar.

Four bacterial strains (*Staphylococcus aureus* ATCC 25923 (SA); methicillin-resistant *Staphylococcus aureus* (MRSA) ATCC 43300, *Escherichia coli* ATCC 25922; *Pseudomonas aeruginosa* ATCC 27853) were used in order to evaluate the effect of AgNPs on bacterial growth rate (GR). For this method, AgACE9 (the tested solution that presented the lowest MIC) was used [6].

### 2.7. Statistical Analysis

The analytical determinations were performed in triplicate. The results were expressed as the mean ± standard deviation (SD). The statistical significance was assessed by GraphPad InStat 3 software (GraphPad Software, San Diego, Canada) at a significance threshold value of *p* < 0.05.

## 3. Results and Discussions

### 3.1. Characterization of Spruce Bark Extract 

The aqueous spruce bark extract was obtained by ultrasound-assisted extraction. TPC was determined from the water extract, phenolics being considered responsible for reducing silver ions, forming nanoparticles, and stabilizing them. Thus, the TPC from SBE was 23.67 ± 1.45 mg GAE/g dw. Literature data provides information about the bioactive aromatic compounds that were identified in the water spruce bark extracts - such as catechin, vanillic acid, gallic acid, etc. [8].

### 3.2. Characterization of AgNPs

**Figure 1:**
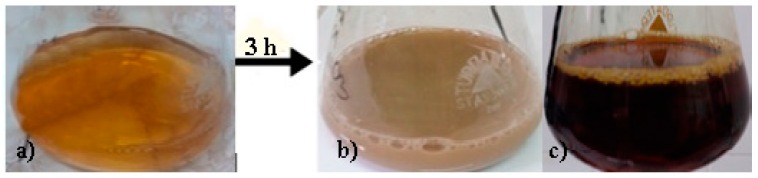
The visual observation of color changes at time 0 (**a**) and after 3 hours for (**b**) AgNIT4 (spruce bark extract, pH = 4, AgNO_3_) and (**c**) AgNIT9 (spruce bark extract, pH9, AgNO_3_).

The color change is an essential factor that confirms the synthesis of AgNPs. When the spruce bark extract was added to the silver nitrate/acetate solution, the color changed from light yellow to opaque gray, at pH 4 (Figure 1a) in brown, at pH 9 (Figure 1b). The final color of the solution confirmed the reduction of silver nitrate/acetate into the AgNPs. Other studies have reported similar color changes, from yellow to gray, as a result of surface plasmon vibrations, which confirm the formation of silver nanoparticles [19,20].

#### 3.2.1. UV-Vis Analysis of AgNPs

**Figure 2:**
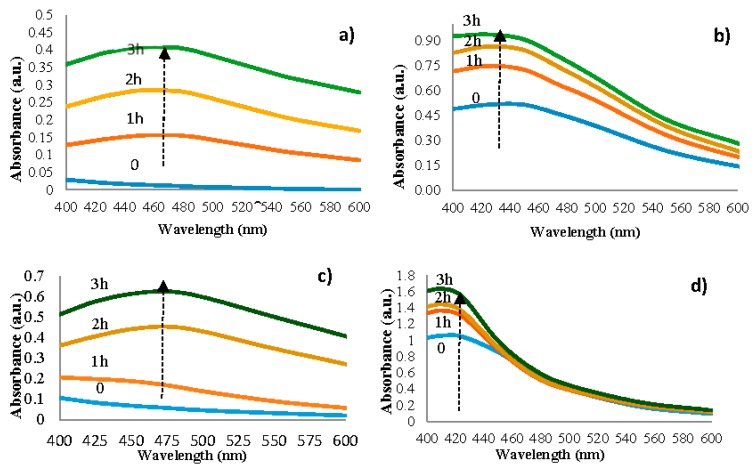
UV-Visible absorption spectra of synthesised silver nanoparticles: (**a**) AgNIT4—spruce bark extract, pH = 4, AgNO_3_; (**b**) AgNIT9—spruce bark extract, pH = 9, AgNO_3_; (**c**) AgACE4—spruce bark extract, pH = 4, AgC_2_H_3_O_2_2; (**d**) AgACE9—spruce bark extract, pH = 9, AgC_2_H_3_O_2_.

The time of biosynthesis, determined visually, is 3 hours for each experimental version, but the time of appearance of the silver nanoparticles is different, and it can be observed by analyzing the UV-Vis spectra (Figure 2).

Silver nanoparticles from AgNIT4 become visible in UV-Vis after maintaining the solution for one hour in the ultrasonic water bath. The absorbance maximum was recorded at the 461 nm wavelength (Figure 2a); these values increase (batochrome displacement) as biosynthesis time passes. It was reported that Ag colloids have absorbance maximum within 400–500 nm, due to Surface plasmon resonance [21]. The silver nanoparticles synthesis, from version AgNIT9, starts from the moment when the solutions are put in contact, and the absorbance maximum was recorded at 431 nm wavelength (Figure 2b). Using the AgC_2_H_3_O_2_ solution (AgACE4), the time of the silver nanoparticles synthesis was 2 hours and the absorbance maximum was recorded at 475 nm wavelength (Figure 2c). AgACE9 silver nanoparticles synthesis starts from the moment when the solutions are put in contact, and the maximum absorbance was recorded at 411 nm wavelength (Figure 2d).

By analyzing the tested solutions comparatively, depending on the used silver solution, no significant differences were found between their absorption spectra.

The influence of pH on AgNPs was investigated by making adjustments at pH 4 and 9. The peak absorbance from the solutions at pH = 9 was higher than at pH = 4. Thus, the increase in the peak intensity of solutions at pH = 9 compared to pH = 4 could indicate a higher AgNPs presence. The higer wavelengths (at pH = 4) indicate an increase in the mean diameter of silver nanoparticles [22], while lower wavelengths (at pH = 9) indicate a decrease of their diameter [23]. These results suggest a significant number of smaller diameter AgNPs when synthesis was driven at pH = 9, and show that AgNPs are present in the colloidal dispersion, stabilized by the bio-compounds from spruce bark extract [24].

#### 3.2.2. FTIR Analysis of spruce bark extract (SBE) and AgNPs

**Figure 3:**
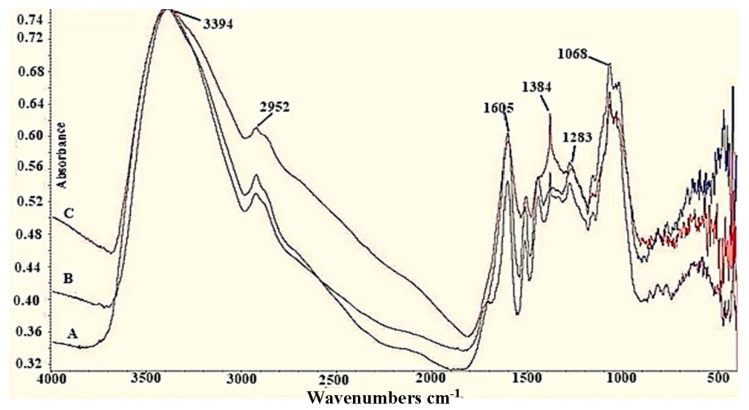
Fourier transform infrared spectra of AgNPs: (**A**)—aqueous spruce bark extract; (**B**)—AgNIT4—spruce bark extract, pH = 4, AgNO_3_; (**C**)—AgNIT9—spruce bark extract, pH = 9, AgNO_3_.

The FTIR spectra of spruce bark extract before and after synthesis of AgNPs are plotted in Figure 3. The spectrum of SBE has several vibration bands indicating the complexity of the extract. Strong bands from wavenumber 3394 cm^−1^ and 1605 cm^−1^ are assigned to the -O-H bonds from phenolic compounds and to the carbonyl group. AgNIT4 and AgNIT9 versions have very well defined, intense and clear bands for -O-H phenolic bonds, -C-H from aldehydes, >C=O, -OH carboxylic, aromatic ethers (3385 cm^−1^, 2931 cm^−1^, 1605 cm^−1^, 1516 cm^−1^, 1070 cm^−1^). In both spectra (AgNIT4 and AgNIT9) an intense band at the wavenumber 1384 cm^−1^ appeared, which corresponds to the asymmetrical valence vibration of the carboxylate ion (COO^-^), a newly formed group after silver reduction (Figure 3). AgNPs spectra, synthesized with silver acetate solution, have the same bands. A synergic action between phenolics and other compounds of spruce bark extract might contributed to the stabilization of NPs [25].

#### 3.2.3. The TEM Analysis of AgNPs

TEM analysis was used to characterize AgNPs in terms of their size and morphology. The results for the different synthesized AgNPs from TEM images were summarized in Table 1. As can be seen, the smaller size (<100 nm) of AgNPs was obtained at pH = 9. The obtained results confirm those presented above (UV-Vis), regarding the basic pH that favors the formation of smaller AgNPs.

**Figure 4:**
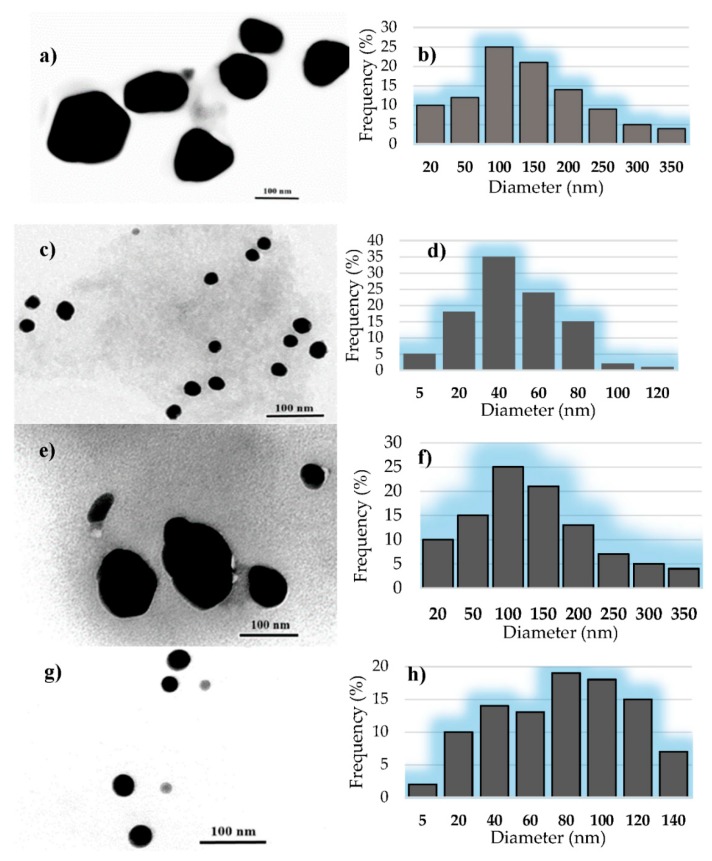
Transmission Electron Microscope (TEM) images and size distribution histogram of silver nanoparticles obtained: TEM image (**a**) and size distribution histogram (**b**) of AgACE4—spruce bark extract, pH = 4, AgC2H3O2; TEM image (**c**) and size distribution histogram (**d**) of AgACE9—spruce bark extract, pH = 9, AgC2H3O2; TEM image (**e**) and size distribution histogram (**f**) of AgNIT4—spruce bark extract, pH = 4, AgNO3; TEM image (**g**) and size distribution histogram (**h**) of AgNIT9—spruce bark extract, pH = 9, AgNO3;.

Figure 4a shows that the silver nanoparticles obtained with silver acetate are nanometric and uniformly (AgACE9) or patchy (AgACE4) distributed. Their morphology varies, meeting spherical and polygonal shapes, and their sizes are between 10–350 nm (Figure 4b). In the AgACE9 version, the spherical shape predominates (Figure 4c), and their sizes are between 10–120 nm; 98% of these have smaller dimensions than 100 nm (Figure 4d). Using silver nitrate in the biosynthesis process, the obtained AgNPs are nanometric and isolately distributed. Figure 4e shows that the morphology varies, and the size of AgNPs is between 10–350 nm; 53% of these have smaller sizes than 100 nm, with an average size 148.64 nm (Figure 4f). For the AgNIT9 version, the morphological and dimensional differences can be observed as a result of a different pH (Figure 4g). Thus, for this tested solution, the obtained AgNPs have sizes between 10–140 nm; 65.15% of these having smaller sizes than 100 nm, with an average size of 75.91 nm (Figure 4h). Literature data shows that silver nanoparticles of various nanosizes and morphologies (spherical, hexagonal, polygonal, etc.) have been synthesized using various stem bark extracts such as *Diospyros montana, Butea monosperma, Holarrhena antidysenterica,* etc. [26,27,28].

### 3.3. Antioxidant Activity

The AgNPs possess free radical scavenging activity against both radicals (DPPH and ABTS), as shown in Table 2. Higher values were noted for AgNIT4 and AgACE4 in comparison with AgNIT9 and AgACE9. The antioxidant activity of AgNPs was evaluated and confirmed by several studies [12,29].

### 3.4. Antibacterial Activity

#### Minimum Inhibitory Concentration 

By analyzing Table 3 it can be observed that AgNPs biosynthesized at basic pH favors the inhibitory activity. AgNPs biosynthesized at pH = 9 (AgNIT9, AgACE9), showed lower MIC values compared to those obtained at pH = 4 (AgNIT4, AgACE4). Also, all AgNPs solutions obtained in the presence of spruce extract showed bactericidal activity, having lower MIC/MBC values than the spruce bark extract.

**Figure 5:**
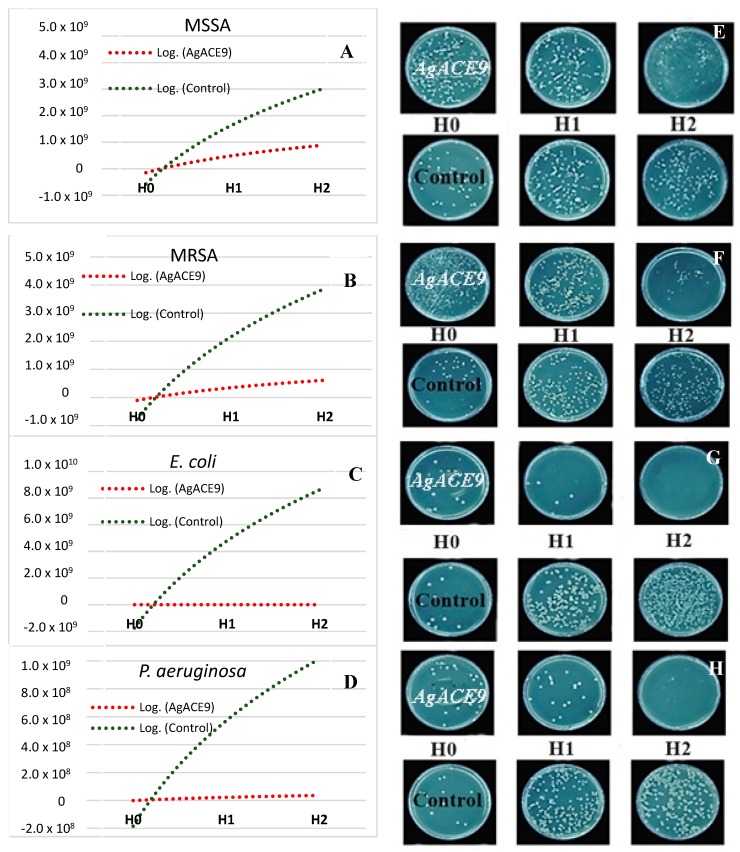
Graphical and visual representation of the growth rate for *Staphylococcus aureus* (**A**,**E**), methicillin-resistant *Staphylococcus aureus* (**B**,**F**), *Escherichia coli* (**C**,**G**), *Pseudomonas aeruginosa* (**D**,**H**) in the presence of AgACE9 (spruce bark extract + AgC_2_H_3_O_2_ pH = 9) and in absence of AgACE9 at: initial time—**H0**, 3 hours—**H1** and 6 hours—**H2**.

As can be seen in the graph and in the visual representation from Figure 5, when spruce bark extract + AgC_2_H_3_O_2_ at pH = 9 were added in the culture medium, the bacterial growth was significantly inhibited within six hours compared to control. At time H 1 (3 h after incubation), AgACE9 inhibited both methicillin-sensitive *S. aureus* and MRSA, and after 6 h of incubation, AgACE9 significantly reduced their bacterial growth. In the case of gram-negative bacteria (*E. coli*, *P. aeruginosa*), after 3 h of incubation, their growth was significantly reduced. At time H2 (6 h after incubation), AgNPs showed a bactericidal effect on *E. coli* and significantly reduced growth of *P. aeruginosa*.

Silver has been demonstrated to have an antibacterial effect [30]. However, silver can be manufactured into AgNPs, for the improvement of its biological properties. Thus, significant antimicrobial activity was confirmed for AgNPs mediated by spruce bark extract and for other AgNPs solutions [13,20,31,32].

Even if there is a significant number of literature data presenting the antibacterial effect of AgNPs, their toxicity has also been recognized [33,34]. Further investigations are needed to elucidate whether, or how AgNPs could be used in patients’ treatments, but until then, there are a lot of other useful applications for the antibacterial effect of these AgNPs (such as water treatment, food industry, sanitization, disinfection, textile industry). Multiple studies support the antibacterial activity of the different types of AgNPs, but due to the differences between the assessed protocols, it is difficult however to effectively compare results. Nevertheless, even if the antimicrobial potential of AgNPs is recognized and strongly supported by literature, it is clear that there is much more information that needs to be clarified in order to completely functionalize these products. For the antibacterial activity, the microdilution method is considered to be superior to the agar diffusion protocol (the method that was chosen by most authors); the first one enables the identification of the MIC values, and with further steps, even the MBC values.

## 4. Conclusions

The present paper describes the biosynthesis of silver nanoparticles mediated by *Picea abies* L. stem bark extract. The spruce bark has bioactive compounds that are responsible for the reduction and capping of Ag ions into AgNPs, thereby providing stability to the silver nanoparticles. Due to the different salt solution or pH, variations in the size of the synthesized AgNPs were observed. The biosynthesized silver nanoparticles have antioxidant activity and intense antibacterial activity against the tested bacteria, probably due to their smaller sizes, obtained for pH9.

Our future research directions will relate to the establishment of practical applications for AgNPs (potentiation of the antibiotic effect, the effect on microbial adhesion on different surfaces, effect on biofilm production), and how these AgNPs will be delivered. The obtained AgNPs could be delivered in patches or hydrogel, and release characterization will be needed. Also, AgNPs could be incorporated into core-sheath structures or as fibre meshes [35,36,37,38].

## Figures and Tables

**Table 1:** Average size and shapes of the AgNPs mediated by spruce bark extract (SBE), obtained from Transmission Electron Microscopy (TEM) micrographs.

Sample Code	Shapes	Average Size (nm)
AgACE4	spherical and polygonal	165.54 ± 8.19
AgACE9	spherical	44.02 ± 0.31
AgNIT4	spherical and polygonal	148.64 ± 11.41
AgNIT9	spherical	75.91 ± 2.77

**Notes:** AgNIT4—spruce bark extract, pH = 4, AgNO_3_; AgNIT9—spruce bark extract, pH = 9, AgNO_3_; AgACE4—spruce bark extract, pH = 4, AgC_2_H_3_O_2_; AgACE9—spruce bark extract, pH = 9, AgC_2_H_3_O_2_. ± Standard deviation.

**Table 2:** Antioxidant Activity of AgNPs.

Sample Code	DPPH mg TE/g of Sample	TEAC mgTE/g of Sample
AgNIT4	11.36 ±0.34	100.37 ± 0.69
AgNIT9	6.91 ± 0.35	29.17 ± 0.35
AgACE4	16.89 ± 0.21	41.03 ± 0.21
AgACE9	2.82 ± 0.15	18.57 ± 0.30

**Notes:** AgNIT4—spruce bark extract, pH = 4, AgNO_3_; AgNIT9—spruce bark extract, pH = 9, AgNO_3_; AgACE4—spruce bark extract, pH = 4, AgC_2_H_3_O_2_; AgACE9—spruce bark extract, pH = 9, AgC_2_H_3_O_2_. ± Standard deviation.

**Table 3:** Antimicrobial activity of AgNPs of tested bacteria.

Pathogenic Bacteria	ATCC No	AgNP Tested Solution	MIC	MBC
			mg/mL	mg/mL
*Staphylococcus aureus*	25923	AgNIT4	1.36	2.27
		AgNIT9	0.05	1.57
		AgACE4	1,24	1.86
		AgACE9	0.14	3.36
		SBE	2.5	2.5
		AgNO_3_	0.02	>0.15
		AgC_2_H_3_O_2_	0.02	>0.15
MRSA	43300	AgNIT4	1.13	1.13
		AgNIT9	0.09	0.25
		AgACE4	1.86	1.86
		AgACE9	0.22	0.70
		SBE	2.5	2.5
		AgNO_3_	0.2	0.2
		AgC_2_H_3_O_2_	0.2	0.2
*Escherichia coli*	25922	AgNIT4	2.73	>2.73
		AgNIT9	0.23	0.31
		AgACE4	>1.86	>1.86
		AgACE9	0.24	0.45
		SBE	>2.5	>2.5
		AgNO_3_	0.2	0.2
		AgC_2_H_3_O_2_	0.2	0.2
*Klebsiella pneumoniae*	700603	AgNIT4	>2.73	>2.73
		AgNIT9	0.63	1.18
		AgACE4	>1.86	>1.86
		AgACE9	1.4	1.96
		SBE	>2.5	>2.5
		AgNO_3_	>0.15	>0.15
		AgC_2_H_3_O_2_	0.03	0.12
*Pseudomonas aeruginosa*	27853	AgNIT4	2.73	>2.73
		AgNIT9	0.16	0.31
		AgACE4	>1.86	>1.86
		AgACE9	0.17	0.84
		SBE	>2.5	>2.5
		AgNO_3_	0.02	0.02
		AgC_2_H_3_O_2_	0.02	0.02

**Notes:** MRSA—methicillin-resistant *Staphylococcus aureus*, AgNIT4—spruce bark extract, pH = 4, AgNO_3_; AgNIT9—spruce bark extract, pH = 9, AgNO_3_; AgACE4—spruce bark extract, pH = 4, AgC_2_H_3_O_2_; AgACE9—spruce bark extract, pH = 9, AgC_2_H_3_O_2_.
